# Efficacy of Antimicrobial and Larvicidal Activities of Green Synthesized Silver Nanoparticles Using Leaf Extract of *Plumbago auriculata* Lam

**DOI:** 10.3390/plants9111577

**Published:** 2020-11-14

**Authors:** Lakshmanan Govindan, Sathiyaseelan Anbazhagan, Ammar B. Altemimi, Karthik Lakshminarayanan, Sivaranjan Kuppan, Anubhav Pratap-Singh, Murugesan Kandasamy

**Affiliations:** 1 CAS in Botany, University of Madras, Guindy Campus, Chennai 600 025, India; lakshmanang261988@gmail.com (L.G.); sathiyaseelan@kangwon.ac.kr (S.A.); profkm68@gmail.com (M.K.); 2 Bharath Institute of Higher Education and Research, Chennai 600 073, India; 3 Food Sciences Department, College of Agiculture, University of Basrah, Basrah 61004, Iraq; 4 ToxiVen Biotech Private Limited, Siva Nagar, Kovaipudhur 641 042, India; karthik@toxiven.com; 5 Department of Chemistry, Faculty of Science and Mathematics, Universiti Pendidikan Sultan Idris, Tanjung Malim 35900, Malaysia; kksivaranjan@gmail.com; 6 Food, Nutrition & Health Program, Faculty of Land and Food Systems, The University of British Columbia, Vancouver, BC V6T 1Z4, Canada

**Keywords:** silver nanoparticles, mosquito repellant, *Plumbago auriculata*, XRD, TEM, antibacterial, larvicidal activity

## Abstract

This work reports the synthesis of silver nanoparticles (AgNPs) using aqueous extract of *Plumbago auriculata*, and evaluates their antibacterial and larvicidal activities. The synthesized silver nanoparticles were characterized by various spectroscopy techniques, such as FTIR, XRD, TEM, EDX, Zeta potential, and DLS. The synthesized AgNPs exhibited significant antibacterial activity against Gram-positive and Gram-negative bacteria, such as *Bacillus subtilis*, *Staphylococcus aureus*, *Escherichia coli,* and *Klebsiella pneumoniae*. Furthermore, synthesized nanoparticles inhibited the fourth instars larvae of *Aedes aegypti* and *Culex quinquefasciatus* at the concentration of 45.1 and 41.1 µg/mL respectively. Results of dose-dependent studies showed that synthesized nanoparticles were also effective at low concentrations. Molecular docking studies performed with the salivary protein and odorant-binding protein of *Aedes aegypti* and *Culex quinquefasciatus* demonstrated that the naphthoquinone compound plumbagin exhibited reliable binding affinity towards the two enzymes. The findings thus reveal that the plant extract and its nanoparticles can be a better alternative to available chemicals to control mosquitos.

## 1. Introduction

Communicable or infectious diseases caused by various human pathogenic microorganisms still remain a global problem in most of the developing and developed countries [1,2,3]. Vector-borne diseases transmitted by mosquitoes that result in malaria, dengue fever, filariasis, ticks, fleas, and other vectors are reported to be endemic in many countries across the world [4,5,6]. In 2018, globally, about 2.5 million cases of malaria were reported, wherein India alone accounted for 76% of the cases [7]. Several millions of humans die from mosquito-borne diseases every year, resulting in a drastic impact on socio-economic development [8,9]. The various synthetic mosquitocidal agents developed to control mosquito population have not only affected humans but also led to the development of resistance towards these synthetic compounds [10,11]. Earlier work on phytochemicals as insecticides and repellents against mosquitoes were promising and encouraged researchers to work further on the development of botanical insecticides [12]. In recent years, the use of nanoparticle and nanoemulsion systems from plant extracts has gained prominent importance due to their significant application in producing compounds exhibiting antimicrobial, anticancer, and antioxidant properties [13,14,15,16]. Eco-friendly green synthesized nanoparticles are safer, low-cost, non-toxic, and thus are considered very useful for pharmaceutical applications facilitating bulk synthesis [17,18,19]. Furthermore, low concentration of silver nanoparticles is found to be non-toxic to eukaryotic cells, including humans, but are known to exert high toxicity against prokaryotic cells of microorganisms, such as bacteria, viruses, and fungi [20].

Currently, several studies have been reported to produce plant-based eco-friendly repellents possessing larvicidal activity [21,22]. These medicinal plants are used for the synthesis of silver, gold, platinum, copper, and titanium nanoparticles having potent antimicrobial, antioxidant, and anticancer properties [23,24]. These medicinal plants naturally possess bio-surfactant molecules, also called secondary metabolites, such as flavonoids, alkaloids, tannins, saponins, glycosides, and phenols. These compounds have been extensively explored for their potency to synthesize silver nanoparticles. The use of environmentally benign materials like plant extracts, bacteria, and fungi for the synthesis of metal nanoparticles offers several benefits, such as eco-friendliness and compatibility for pharmaceutical and other biomedical applications, as they do not use toxic chemicals [25,26].

*Plumbago auriculata* Lam. (Plumbaginaceae) is one such plant that has been reported to possess various secondary metabolites in their leaves [27,28]. To the best of our knowledge and literature survey, *Plumbago auriculata* Lam. has not been used for the synthesis of silver nanoparticles (AgNPs). Therefore, in this study, an attempt was made to perform green synthesis of AgNPs using *Plumbago auriculata* Lam. Synthesized AgNPs were characterized to assess their antibacterial activity against four microbial strains. Following this, their larvicidal activity towards the fourth instar larvae of *Aedes aegypti* (*Aedes aegypti*) and *Culex quinquefasciatus* (*Culex quinquefasciatus*) were also investigated. Finally, molecular docking studies were performed with a naphthoquinone-derived compound ‘plumbagin’, which is known to be predominantly present across the Plumbaginaceae family of plant species [29,30]. Using Auto Dock 4.2, attempts were made to dock plumbagin with the crystal structures of D7 salivary protein and odorant-binding protein (OBP) from *Aedes aegypti* and *Culex quinquefasciatus*, respectively. The docking results showed that plumbagin possessed reliable hydrogen bond interactions with high dock scores compared with the respective co-crystal ligands of the target enzymes.

## 2. Results and Discussion

### 2.1. Characterization of AgNPs Synthesized Using Plumbago auriculata Lam

**Figure 1:**
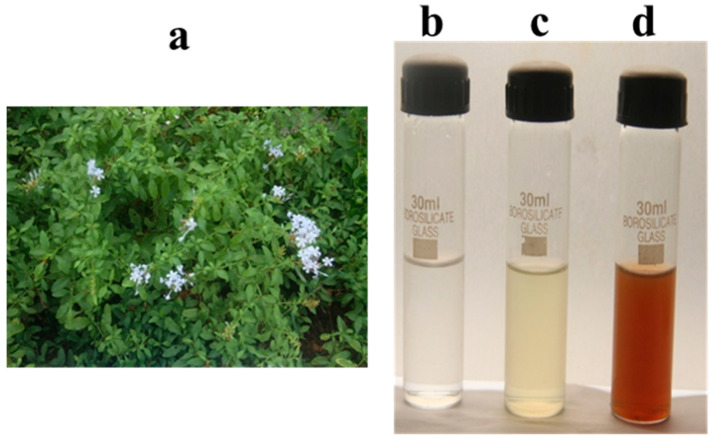
Synthesis of silver nanoparticles using leaf extract of *Plumbago auriculata* Lam.—(**a**), AgNO3—(**b**), aqueous extract—(**c**), synthesis of PA-AgNPs—(**d**).

The secondary metabolites present in the *Plumbago auriculata* Lam. plant extract act as reducing and capping agents and thus significantly facilitate the formation of silver ions from silver nitrate for the synthesis of AgNPs. Similar studies showed the presence of secondary metabolites, such as flavonoids, alkaloids, steroids, phenols, tannins, and other bioactive metabolites, that are mainly responsible for the synthesis of AgNPs [9]. After 30 min of incubation, the reaction mixture changed from a clear transparent pale yellow to dark brown (Figure 1a–d), due to the characteristic surface plasmon resonance (SPR) property exhibited by the metal nanoparticles, amongst other factors that influence the color of nanoparticles like concentration, size, etc.

**Figure 2:**
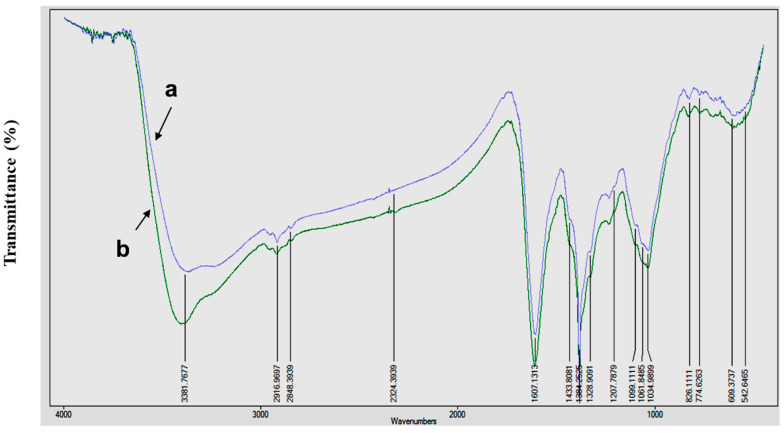
FTIR spectral analysis of PA-AgNPs –(**a**), and PA leaves extract –(**b**).

FTIR analysis was carried out to identify the possible reducing and capping biomolecules in the synthesized AgNPs. The spectra of AgNPs revealed strong bands at 3381 (due to primary amines), 2916 (C-H stretching of the alkanes), 2848 (C-H-O stretching of aldehyde), 1607 (N-H bend of primary amines), and 1207 cm^−1^ (C-N stretching of aliphatic amines) [26]. Apart from this, the FTIR spectra detected bands at 1384 cm^−1^, corresponding to the presence of stretching vibrations of alcohol, ethers, esters, carboxylic acids, and amino acids [31], and 1034 cm^−1^ (C-O stretching of alcohol), which is related to the secondary metabolites present in the aqueous extract of *Plumbago auriculata* Lam. The spectral data also confirmed the presence of proteins by amine or amide I band at the region of 1607 cm^−1^ (Figure 2). Similar FTIR spectrum peaks (CHO, CN, NH, CH, and CO) were observed in *Caesalpinia pulcherrima* [32] and *Cacumen platyclade* [33], where AgNPs were synthesized in the plant extracts. It has been also reported that the presence of secondary metabolites helps in maintaining the stability of AgNPs and also prevents aggregation [34].

**Figure 3:**
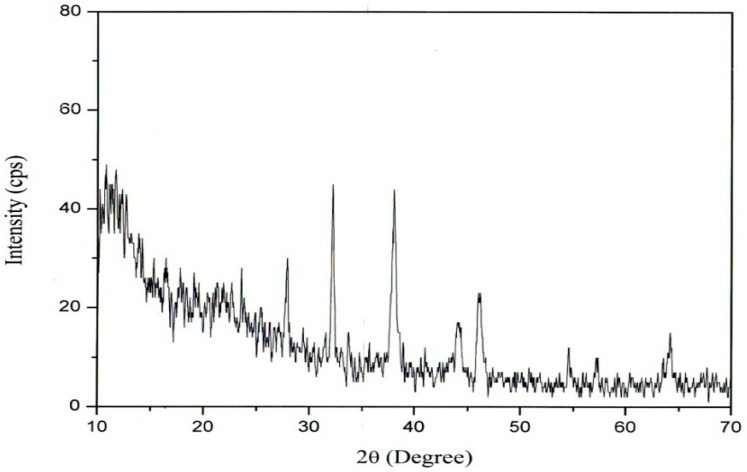
XRD pattern of synthesized PA-AgNPs.

**Figure 4:**
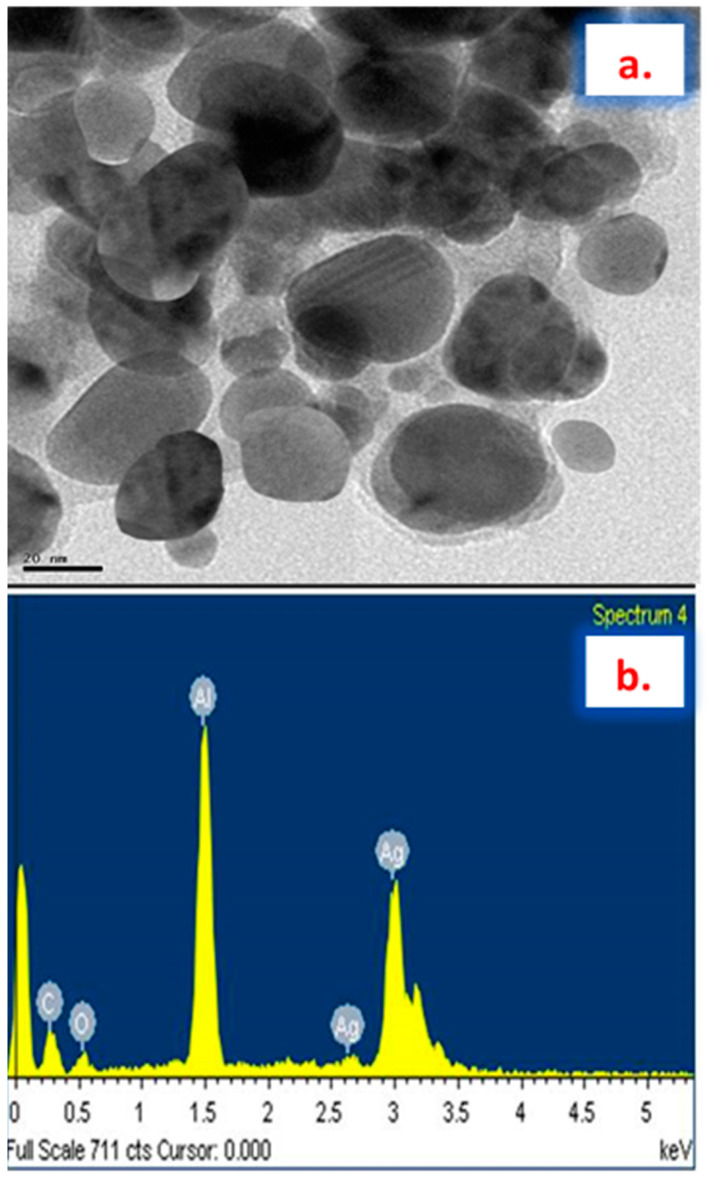
(**a**) TEM and (**b**) EDX images of the AgNPs synthesized using *Plumbago auriculata* Lam. extract.

The synthesized AgNPs from *Plumbago auriculata* Lam. were further confirmed by X-ray diffraction pattern analysis. Results showed strong diffraction peaks in the spectrum of 2θ values ranging from 10° to 70° (Figure 3). The peaks correlated with a face-centered cubic (fcc) crystal structure indexed by Bragg’s reflections corresponding to the (111, 200, 220, 311) plane. Some noise peaks were also observed, which could be due to the presence of organic material capped on the silver. Strong signals found in the EDX analysis (Figure 4b) confirmed the presence of silver nanoparticles. Similarly, the occurrence of weak signals from the same analysis confirmed the presence of some organic molecules, which is similar to earlier reports [35]. 

The synthesized PA-AgNPs were observed under TEM and are shown in Figure 4a. The shape of the particles was shown to be primarily spherical and hexagonal with a particle size of <50 nm. Further, the TEM image of PA-AgNPs indicated smaller particles without agglomeration due to the phyto-molecules capping on the surface of the nanoparticle. The above findings followed earlier reports where plant extract was employed in synthesis of AgNPs [36,37]. Thus, the synthesized AgNPs were found to depend upon the presence of secondary metabolites, such as phenolic compounds, flavonoids, steroids, tannins, and others, indicating that they might have played a vital role in the synthesis of AgNPs.

**Figure 5:**
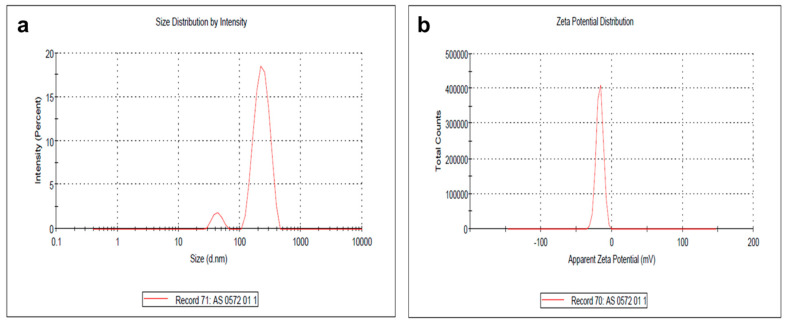
Particle size distribution—(**a**); and zeta potential analysis of PA-AgNPs—(**b**).

DLS analysis showed that the synthesized PA-AgNPs were polydispersed with a particle size of 20 to 500 nm (Figure 5a). The size of the synthesized AgNPs was found to agree well between the TEM and DLS analysis. This result indicated that plant components were effectively involved in the synthesis and controlled formation of silver nanoparticles. Furthermore, surface zeta potential is one of the essential parameters for characterizing the stability of nanoparticles in water as a dispersant. It can be used for long-term stability prediction, wherein it measures the magnitude of electrostatic charge repulsion or attraction between the particles in a liquid suspension. The stability of the nanoparticles was shown at −17.1 mV (Figure 5b). These high negative values specified good stability, well-dispersed nature, and possible presence of secondary metabolites as a capping or reducing agent for nanoparticles [13,38]. Results were consistent with the literature studies, where the zeta potential value for AgNPs synthesized from Coptis chinensis was found to be −30 mV and remained stable [23].

### 2.2. Antibacterial Activity

**Figure 6:**
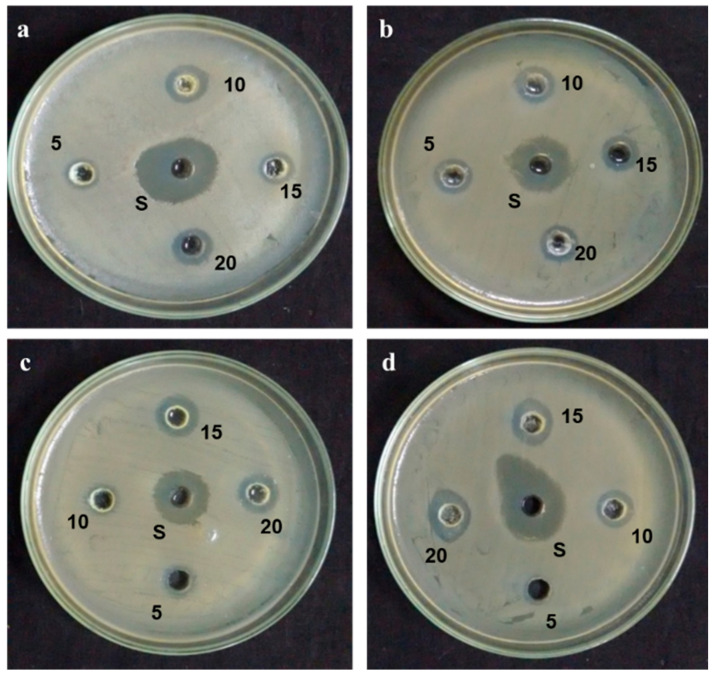
Antibacterial activity of green synthesized AgNPs. *Bacillus subtilis*—(**a**); *Staphylococcus aureus*—(**b**); *Escherichia coli*—(**c**); *Klebsiella pneumoniae*—(**d**).

In this study, synthesized AgNPs from the aqueous extract of *Plumbago auriculata* Lam. exhibited significant antibacterial activities against Gram-positive and Gram-negative bacteria, such as *Bacillus subtilis, Staphylococcus aureus, Escherichia coli*, and *Klebsiella pneumoniae*. The maximum zone of inhibition for synthesized AgNPs was observed in *Staphylococcus aureus* (10 ± 1.5 mm), *Escherichia coli* (12 ± 2.5 mm), *Bacillus subtilis* (8 ± 1.0 mm), and *Klebsiella pneumoniae* (14 ± 1.7 mm), respectively, with concentrations of 20 µg/mL. The zone of inhibition results confirmed that the synthesized AgNPs exhibited significant antibacterial activity as compared with aqueous extract of *Plumbago auriculata* Lam. as well as AgNO_3_ (Figure 6 and Table 1). The results suggested that plant-mediated synthesis of silver nanoparticles is capable of producing AgNPs with capability of interaction with the negatively charged surface of the bacterial cell membrane, that thereby inhibits bacterial growth. Hence, plant-synthesized AgNPs could be used as an alternative source of antibiotics against microbial pathogens [39].

### 2.3. Larvicidal Activity of Synthesis AgNPs

The synthesized AgNPs exhibited potent larvicidal activity against *Aedes aegypti* and *Culex quinquefasciatus* towards the fourth instars exposed in different concentrations from 20–100 µg/mL at 24 h. The maximum mortality was observed with LC_50_ values of 45.1 and 41.1 µg/mL against *Aedes aegypti* and *C. fasciatus* respectively(Table 2). The lowest concentration (20 µg/mL) of AgNPs synthesized from plant extracts showed no mortality after 24 h. The mortality rate was significantly increased with an increasing dose-dependent manner where the highest mortality rate was observed with increasing concentrations of synthesized AgNPs. This difference in lethal concentrations might be due to differences in secondary metabolites present in the plant. This was in concordance with the previous reports exploring the insecticidal activity of the phytochemicals through the dose-dependent response [40].

### 2.4. Molecular Docking-Mosquito Salivary Proteins 

In the present study, the larvicidal activity of *Plumbago auriculata* Lam. was explored by the use of AgNPs. As seen from previous reports [29,30], the naphthoquinone compound plumbagin is found to be predominantly present in the Plumbaginaceae plant family. In particular, Maniafu et al. [29] reported the presence of two compounds, viz. 5-hydroxy-2-methyl-1,4-naphthoquinone (plumbagin)’ and β-sitosterol, in the crude extracts from three *Plumbago* spp. *Plumbago zeylanica* (*P. zeylanica*) Linn, *Plumbago dawei* (*P. dawei*) Rolfe, and *Plumbago stenophylla* Bull. They attributed plumbagin to be primarily responsible for the larvicidal activity against *Anopheles gambiae*. In a similar work, the root compound of plumbagin from *Plumbago zeylanica* was evaluated for antimalarial activity against the fourth instar larvae of *Anopheles stephensi* [30]. In 2014, Deshpande et al. [41] reported the presence of plumbagin in the leaves of *Plumbago auriculata* Lam. Hence, based on these observations, the present study focused on performing molecular docking of plumbagin with the mosquito salivary proteins. 

**Figure 7:**
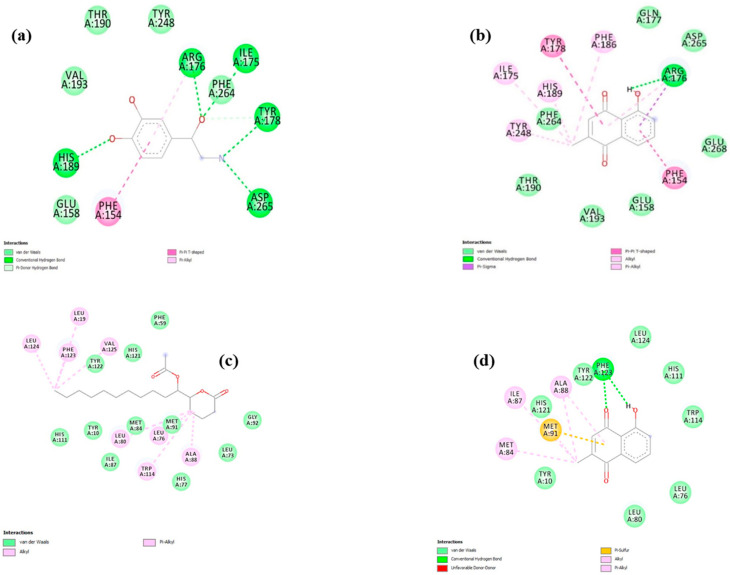
Docking analysis of co-crystal epinepheine (**a**) and plumbagin (**b**) with 3DXL; co-crystal acetate (**c**) and plumbagin (**d**) with 3OGN.

The binding mode and interactions of plumbagin with D7 salivary protein of *Aedes aegypti* and OBP of *Culex quinquefasciatus* were examined by molecular docking studies. The mosquito D7 salivary proteins are known to be functionally related to the superfamily of arthropod odorant-binding proteins (OBPs). In recent times, the mosquito salivary glands have been explored for identifying the key proteins that facilitate blood-feeding and can be considered potential targets for malaria transmission-blocking interventions. The results showed that plumbagin exhibited a docking score of −6.71 (kcal/mol) and inhibitory constant of 12.1 uM against D7 protein of *Aedes aegypti*, which were relatively higher than the docking score and inhibitory constant obtained for co-crystal ligand epinephrine (Table 3). Nevertheless, both compounds possessed reliable hydrogen bond interactions with the active site key residues (Table 3 and Figure 7a,b). Similarly, molecular docking of plumbagin with OBP showed that it exhibited a docking score of −7.48 (kcal/mol) and an inhibitory constant of 3.31 uM (Table 3). Though the values were found to be relatively less than the values obtained for co-crystal ligand, still hydrogen bond interactions were observed only for plumbagin (Table 3 and Figure 7d). Collectively, the docking results suggest that plumbagin possesses a relatively higher binding affinity towards the D7 salivary protein of *Aedes aegypti* and a comparable binding affinity towards OBP of *Culex quinquefasciatus*. The outcome of these results suggests that plumbagin, from *Plumbago auriculata* Lam., can be developed into a potent antimalarial and antifilarial compound in the treatment of several vector-borne diseases.

## 3. Materials and Methods 

### 3.1. Materials 

AgNPs were synthesized from *Plumbago auriculata* Lam. aqueous extract; silver nitrate (AgNO_3_) was purchased from Sigma Aldrich; nutrient agar (NA) and Muller Hilton Agar (MHA) were procured from HiMedia, India; and dimethyl sulfoxide (DMSO) from SD Fine-Chem Limited, India.

### 3.2. Preparation of Plumbago auriculata Lam. Aqueous Extract

Fully grown leaves of *Plumbago auriculata* Lam. were collected from the University of Madras, Guindy Campus, and initially cleaned using running tap water followed by double-distilled water to remove the dust particles. Around 10 g of washed leaves were boiled in 100 mL of distilled water for 30 min at 60 °C. Using Whatman filter paper, the aqueous crude extract was retained and used for further experiments.

### 3.3. Synthesis of AgNPs Aqueous Extract Using P. auriculata

The obtained (10 mL) aqueous extract was incubated with 1 mM of silver nitrate solution (100 mL) in a volumetric flask in the dark condition at 24 h, as plant extracts containing phenolics are known to be susceptible to photodegradation [42]. The synthesis of AgNPs was done at room temperature (25 °C ± 2 °C). The change in the color of the solution after 24 h from light green to brown indicated the reduction of AgNPs. 

### 3.4. Characterization of AgNPs

The green synthesized AgNPs were characterized by X-ray diffraction (XRD), used to investigate the crystalline structure of AgNPs. XRD was recorded in the 2θ range (30–80) using XRD6000 (Shimadzu). A transmission electron microscope (TEM) was used to determine the size and shape of AgNPs. The grid was left to dry overnight at room temperature before TEM analysis. The presence of functional groups in *Plumbago auriculata* Lam. extract-synthesized AgNPs were identified by a Shimadzu 8400 FTIR Spectrophotometer (Perkin Elmer Spectrum) using the KBr pellet technique at the range of 4000–400 cm^−1^. The morphology of AgNPs was examined using FESEM, and the presence of silver was confirmed by EDX. The average size of AgNPs and their stability was determined by dynamic light scattering (DLS, Malvern Instruments Ltd., Malvern, UK) according to the methods previously reported [13,26,38].

### 3.5. Antibacterial Activity

The antibacterial activity of synthesized AgNPs was determined by the well diffusion method against *Bacillus subtilis* (*B. subtilis*), *Staphylococcus aureus* (*S. aureus*), *Escherichia coli* (*E. coli*), and *Klebsiella pneumoniae* (*K. pneumoniae*) on Muller-Hinton agar plates. The incubated bacterial culture (10^7^/mL) was swabbed uniformly using a sterile cotton swab. Various concentrations of (5–20 µg/mL) silver nanoparticles were poured into each well on all the plates after which they were incubated at 37 °C for 18 h. After incubation, the clear zone appeared, and it was measured as a zone of inhibition. Standard antibiotic tetracycline was used as a positive control [43].

### 3.6. Collection of Mosquitos’ Larvae

The fourth instar larvae of *Aedes aegypti* and *Culex quinquefasciatus* were collected from a drainage, septic tank, and polluted water area of Tambaram, and they were identified by Entomology Research Institute at Loyola College in Chennai, Tamil Nadu, India. The larvicidal activity was conducted using the general procedure followed by the World Health Organization, 1998. They were collected and reared in the laboratory as per Deshpande et al. [41]. The larvae were maintained in a water-containing plastic tray with sufficient feed at the condition of 25–27 °C and 75–85% humidity and the egg rafts were collected from each cage to maintain the next generation.

### 3.7. Bioassay for Larvicidal Activity

The synthesized AgNPs were used at various concentrations (20–100 µg/mL). Each test included a set of control groups (distilled water) with five replicates for each concentration. The dose–response data were subjected to probit analysis to determine the LC_50_ values at 24-h exposure under constant climatic conditions. Larvae were added to 249 mL of water and 1.0 mL of prepared plant extract by maintaining five replicates. The control WAS maintained without extract and the replicates were left for exposure for 24 h. Then, the number of viable larvae was reported for assessment of the mortality rate from the mean of triplicates using Equation (1). The extracts showing higher activity were considered for further studies:

(1) $$\mathrm{Percentage}\;\mathrm{of}\;\mathrm{mortality}=\frac{\mathrm{No}\;\mathrm{of}\;\mathrm{dead}\;\mathrm{larva}}{\mathrm{No}\;\mathrm{of}\;\mathrm{larva}\;\mathrm{introduced}}\;\times 100.$$

### 3.8. In Silico Docking Study

A molecular docking study was performed to determine the binding efficacy of plumbagin in the active site of the chosen drug targets. A Lamarckian genetic algorithm method, implemented in the Auto Dock 4.2 program, was employed [44]. The ligand compound plumbagin was retrieved from the PubChem database. The ligand free form of D7 Salivary Protein of *Aedes aegypti* (PDB:3DXL) and OBP of *Culex quinquefasciatus* (PDB: 3OGN) was fetched from the Protein Data Bank. Before molecular docking, using the PRODRG server, energy-minimized 3-D atomic coordinates of plumbagin was generated [45]. Following this, Gasteigere–Marsili partial charges were assigned to plumbagin and nonpolar hydrogen atoms were merged. All torsions were allowed to rotate during docking. A grid box of size 50 Å × 50 Å × 50 Å with a spacing of 0.375 Å was prepared at the active site of both the enzymes (based on the hydrogen bonding interactions exhibited by their respective co-crystal ligands). The Lamarckian Genetic Algorithm (LGA) was used for molecular docking, where a maximum of 15 conformers was considered for each compound. Using Auto Dock 4.2, molecular docking was compiled and run under Microsoft Windows XP operating system.

### 3.9. Statistical Analysis

All data were expressed as mean ± standard error. The average larval mortality data were subjected to probit analysis to calculate the LC_50_ (lethal concentrations) values. Their statistics at the 95% upper confidence limit (UCL) and lower confidence limit (LCL) values were estimated by fitting a probit regression model. All the analyses were calculated using the Statistical Package of Social Sciences (SPSS) software package version 16.0. Results with *p* < 0.05 were considered to be statistically significant.

## 4. Conclusions

In the present study, we successfully synthesized AgNPs using aqueous *Plumbago auriculata* Lam. plant extract. Several characterization studies, such as FTIR, TEM, XRD, EDX, zeta potential, and DLS, were performed to evaluate the synthesized AgNPs. AgNPs showed good stability, with a particle size ranging from 20 to 500 nm. The synthesized AgNPs from *Plumbago auriculata* Lam. extract showed a significant antibacterial activity against various species. The capping of plant constituents, such as secondary metabolites, on the surface of AgNPs might have enhanced this activity. Apart from exerting antibacterial property, the synthesized AgNPs from the plant extract also exhibited potent larvicidal activity against the fourth instar larvae of *Aedes aegypti* and *Culex quinquefasciatus*. The molecular docking studies performed with the salivary protein and odorant-binding protein of *Aedes aegypti* and *Culex quinquefasciatus* respectively showed that the naphthoquinone compound plumbagin exhibited reliable binding affinity towards the two enzymes. Collectively, these results suggest that the synthesis of metal nanoparticles by plant extracts can be optimized further in such a way that nanoparticles of desired quality and activity can be produced. Such eco-friendly nanoparticles can be efficiently used in bactericidal, wound healing, and targeted drug delivery systems, making this method potentially useful in large-scale production of similar metallic nanomaterials.

## Figures and Tables

**Table 1:** Antibacterial activity of PA-AgNPs.

	*Bacillus subtilis*	*Staphylococcus aureus*	*Escherichia coli*	*Klebsiella pneumoniae*
PA-AgNPs (μg/mL)	Zone of inhibition (mm)			
5	8 ± 0.5	10 ± 1.5	10 ± 0.8	11 ± 0.5
10	10 ± 1.7	10 ± 0.8	10 ± 0.6	11 ± 0.8
15	8 ± 0.9	8 ± 0.7	12 ± 1.0	12 ± 1.0
20	8 ± 1.0	10 ± 1.5	12 ± 2.5	14 ± 1.7
Streptomycin (20)	18 ± 2.0	15 ± 2.8	15 ± 3.7	18 ± 1.5
	Minimum inhibitory concentration (MIC)			
PA-AgNPs (μg/mL)	10 ± 0.5	6 ± 0.5	8 ± 0.5	10 ± 0.8

**Table 2:** Larvicidal activity of synthesized AgNPs using *Plumbago auriculata* Lam. against *Aedes aegypti* and *Culex quinquefasciatus.*

**Species**	**Concentration (µg/mL)**	**Mortality (24 h)**	**LC_50_** **(µg/mL)**	**LUL-UCL** **(µg/mL)**	**r^2^**	**Regression Equation**
*Aedes aegypti*	100	95.0	45.1	37.4 −54.3	0.986	Y = 0.787X + 15.9
	80	80.7				
	60	61.0				
	40	44.0				
	**20**	34.7				
**Species**	**Concentration (µg/mL)**	**Mortality (24 h)**	**LC_50_** **(µg/mL)**	**LUL-UCL** **(µg/mL)**	**r^2^**	**Regression Equation**
*Culex quinquefasciatus*	100	91.0	41.1	34.6–48.9	0.997	Y = 0.785X + 15.8
	80	83.5				
	60	65.0				
	40	41.5				
	20	33.5				

**Table 3:** Molecular docking of plumbagin with D7 salivary protein of *Aedes aegypti* and odorant-binding protein of *Culex quinquefasciatus.*

Sl. No	Compound	Dock Score (kcal/mol)	Inhibitory Constant	H Bond Interactions (H-D…A)	Distance (Å)
	Plumbagin with 3DXL (*Aedes aegypti*)				
1	Co-Crystal (Epinephrine)	−5.80	55.64 uM	O-H…O Ile 175 Tyr 178 O-H…N His 189 N(E2)-H…O	3.1 2.9 3.4
2	Plumbagin	−6.71	12.1 uM	O-H…O Arg 176 His 189 N(E2)-H…O	2.7 3.1
Plumbagin with 3OGN (*Culex quinquefasciatus*)					
3	Co-Crystal (Hexadecanolide)	−7.82	1.84 uM	--	--
4	Plumbagin	−7.48	3.31 uM	PHE 123 N-H…O O-H…O PHE 123	2.7 2.8
